# Total Phenolic Content and Antioxidant and Antibacterial Activities of *Pereskia bleo*

**DOI:** 10.1155/2019/7428593

**Published:** 2019-01-02

**Authors:** Mas Athira Johari, Heng Yen Khong

**Affiliations:** Faculty of Applied Sciences, Universiti Teknologi MARA, 94300 Kota Samarahan, Sarawak, Malaysia

## Abstract

Different solvent extracts of *Pereskia bleo* leaves were evaluated for total phenolic content (TPC) and antioxidant activities based on the Folin–Ciocalteu test and DPPH scavenging activities. The antibacterial activities against four bacteria, namely, Gram-positive bacteria: *Streptococcus pyogenes* ATCC 19615 (SP) and *Staphylococcus aureus* ATCC 29737 (SA) and Gram-negative bacteria: *Escherichia coli* ATCC 10536 (EC) and *Pseudomonas aeruginosa* ATCC 9027 (PA), were also performed based on the minimum inhibitory concentration (MIC) and minimum bactericidal concentration (MBC) assays. The findings demonstrated that both the methanolic and chloroform extracts displayed strong activities against SA, SP, EC, and PA while the hexane extract demonstrated the weakest activities towards all the four bacteria. The methanolic extract also exhibited higher TPC and possessed higher antioxidant activity with the IC_50_ value 33.83 *µ*g/mL compared to the chloroform and hexane extracts. As such, the methanolic extract has a higher ability to scavenge free radical compared to other extracts. Due to the interesting result, activities are shown by the methanolic and chloroform crude extracts of *P. bleo*; hence, the study has been extended to the isolation of bioactive compounds to uncover its great potential as a natural source for antibacterial and antioxidant agents.

## 1. Introduction

Medicinal plants have been used to promote human health, and it has been documented since ancient times. Even though accessibility to modern healthcare nowadays become faster and easier, still there are people who prefer to promote health by using fresh medicinal plants which are locally grown. For example, in the way to compliment the allopathic medicine, Malays prefer Jamu medicine and Chinese prefer Traditional Chinese medicine (TCM) while Indians prefer Ayurvedic medicine. Therefore, even though there is an abundance of modern medicine in the market, traditional medicine still maintained its popularity in the developing world.

*P. bleo* is well known as the medicinal plant of the Cactaceae family, which consists of 100 genera and about 2000 species [1]. The origin of the genus *Pereskia* is from South America, and they have been cultivated in tropical countries [2] such as Malaysia, Indonesia, Singapore, and India. Some *Pereskia* species are used as traditional medicines to treat hypertension, diabetes, cancer, high blood pressure, skin inflammation, and skin injuries. Previous reports revealed that the preparation methods of this plant varied subject to the uses. For example, a decoction from the leaves is prepared and then used as a warm bath to alleviate muscle ache while the leaves are boiled for making tea and then drinking it warm or cold for prevention of cancer and detoxification of the body.

Previous phytochemical studies of *Pereskia* genus have revealed the occurrence of a variety of compounds including carotenoids, alkaloids, flavonoid, lactone, sterols, terpenoids, fatty acids, phytosterol glycoside, and phenolic compounds [1]. Besides, studies of *Pereskia* species on its biological activities have been reported on antioxidant, anticancer, antinociceptive, and antibacterial. Moreover, previous phytochemical studies of *Pereskia* genus have revealed the occurrence of a variety of compounds. Although there are many reviews on the traditional uses for a variety of prophylactic and therapeutic purposes, only few *Pereskia* species have been investigated on its phytochemical and biological activities. Therefore, *P. bleo* is selected to further study on its biological activities.

The intention of this study is to explore the biological activities of the *P. bleo* crude extract in exploring the potential of natural sources for the drug discovery which can be used for further innovation development of pharmaceutical, medicinal, health, and household products in order to increase its commercial values. Studies on medicinal plants not only discover new therapeutics but also assist in setting appropriate guidelines and policy in the usage of the traditional herbal medicine since there are scientific evidence and proper understanding of traditional usage of the plants available.

## 2. Materials and Methods

### 2.1. Plant Material

The studied plant sample which is the leaves of *P. bleo* was collected from Perlis, Malaysia. The sample was air-dried at a room with room temperature and good air ventilation. A dried sample was cut into smaller pieces to make it easier for it to be grounded using an electric cutting mill to produce fine powder sample.

### 2.2. Extraction

The air-dried leaves (926 g) of *P. bleo* were extracted at 24 hours interval by the cold extraction method, and the process was repeated twice using methanol as a solvent. Solvents removal under diminished pressure using a BUCHI model R-200 rotavapor produces 39.1371 g dark-green methanol crude extracts. Chlorophyll was removed from the methanol extract before being performed the fractionation. The fractionation process which was performed using liquid-liquid partition have yielded 0.334 g green hexane fraction, 5.8916 g dark-yellow chloroform fraction, and 25.3989 g dark-brown methanol fraction.

### 2.3. Total Phenolic Content

The Folin–Ciocalteu test was chosen to measure TPC of *P. bleo* extracts. This test was performed by referring to the method developed by Velioglu et al. [3] with some modifications. The crude sample was prepared by liquefying 10 mg of the extract in 10 mL of the solvent to yield a concentration of 1 mg/mL. About 100 *µ*L of the extract (1 mg/mL) was combined and mixed with 0.75 mL of the Folin–Ciocalteu reagent (diluted 10-fold with deionized water previously) in the test tube. The liquid mixture was allowed to stand for 5 minutes at a room temperature. The mixture was then added about 0.75 mL of sodium carbonate (Na_2_CO_3_), and the test tube was shaken gently to mix them. After 90 minutes, the absorbance of the mixture was measured using the UV-Vis spectrophotometer at 725 nm.

A calibration curve of standard reference was established using gallic acid (range of concentration from 0.01 to 0.05 mg/mL) as standard references plotted. TPC was revealed as gallic acid equivalents in milligrams per 100g of the extract.

### 2.4. Antioxidant Activity

The DPPH was used to determine free radical scavenging activity as previously described by Shimada et al. [4]. About 3.94 mg of DPPH was first dissolved in 100 mL of ethanol to a concentration of 0.1 mL. About 1 mL of DPPH solution was added to 3 mL of the samples with different concentrations (250, 125, 62.5, 31.25, and 15.62 *μ*g/mL).

For the control test, the same amount of ethanol was added. All the mixture was mixed well by shaking vigorously and left to stand for 30 minutes at a room temperature. After that, the UV-Vis spectrophotometer was used to measure the value of absorbance of each mixture at 517 nm. The calculation for the percentage of inhibition (*I*%) of the DPPH radical is as follows:

(1) $$\begin{array}{c} I\%\;\;=\;\;\left[\left(\frac{A_{\text{o}}\;\;-\;\;A_{\text{s}}}{A_{o}}\right)\right]\times 100\%, \end{array}$$

where *A*_S_ represents the absorbance value of the sample while *A*_0_ represents the absorbance value of the control reaction (contain all reagents except the sample). A graph of inhibition percentages (*I*%) against concentrations of the sample was plotted. From the graph, 50% inhibition (IC_50_ value) provides the value of concentrations for each sample. All experiments were carried out in triplicate to minimize the precision error. Mean ± SD of triplicates was reported as IC_50_ values.

### 2.5. Antimicrobial Activity

Antimicrobial activities were conducted using MIC and MBC assays. The MIC method as described by Gulluce et al. [5] involves the broth microdilution technique using 96-well microplates. In this study, four types of bacteria are used which are SP, SA, EC, and PA.

### 2.6. Minimum Inhibitory Concentration

The MIC represents the lowest concentration that had absence of microscopically noticeable growth. The interpretation of *in vitro* data is based on attainable sample concentrations. Nutrient broth (NB) was prepared as the medium. About 3.6 mg crude extract samples were weighted and dissolved in 2 mL DMSO (stock solution, 1800 *μ*g/mL). Inoculations of the microbial strains were produced from 24 hours broth cultures and suspensions regulated to 0.5 McFarland standard turbidity. About 100 *µ*g/mL sterile NB was added into 96-well plate in rows B to H. After that, about 100 *µ*g/mL of the stock solution was added into rows A and B. Well-mixed mixture of sample and NB at row B were shifted to each well in order to achieve a two-fold serial dilution of samples (1800, 900, 450, 225, 112.5, 56.25, 28.13, and 14.07 *μ*g/mL). Lastly, about 100 *µ*g/mL incubated bacteria were added to all wells (from A to H). The 96-well plate was capped with a lid, airtight, and incubated for 24 hours at 37°C. The microbial growth was recognized by turbidity and the presence of pallet at the base of the wells.

### 2.7. Minimum Bactericidal Concentration

MBC was used to reconfirm the results of MIC by determining the number of the surviving organism through observing the growth of the bacteria. MBC represents the concentration at which 99% of the bacteria were killed. About 10 *μ*g/mL of the solution in the 96-well plate (from MIC analysis) at the first clear stage was transferred on the Petri dish containing nutrient agar (NA) using micropipette and spread using sterilized cotton bud. The Petri dish was incubated for 24 hours at 37°C. If the NA inside the Petri dish is clear during observation, meaning no bacteria growth, so the MBC result is the same as the MIC result. Meanwhile, if the NA turns cloudy or not clear, the MBC result showed that concentration is one step higher than MIC. The agar was prepared by dissolving NA powder (20 g/L) in distilled water and then mixed. The mixture was autoclave at 121°C for 15 minutes. When the NA solution is warm enough, the media is poured aseptically into Petri dishes.

## 3. Results and Discussion

### 3.1. Total Phenolic Content

TPC activity is the process to figure out the amount of phenolic content in the samples. Phenolic compounds that contained in the plants have redox properties, and the properties allow them acting as antioxidants [6, 7]. The results (Table 1) showed that the methanolic extract exhibited higher TPC as compared to the chloroform and hexane extracts which are approximately about 40.82 mg GAE/g for methanolic extract, 31.91 mg GAE/g for chloroform extract, and 25.2 mg GAE/g for hexane extract. Higher phenolic content in the methanolic extract is responsible for bioactivity; therefore, this extract is expected to exhibit good result in antioxidant and antibacterial activities.

### 3.2. Antioxidant Activity

**Figure 1:**
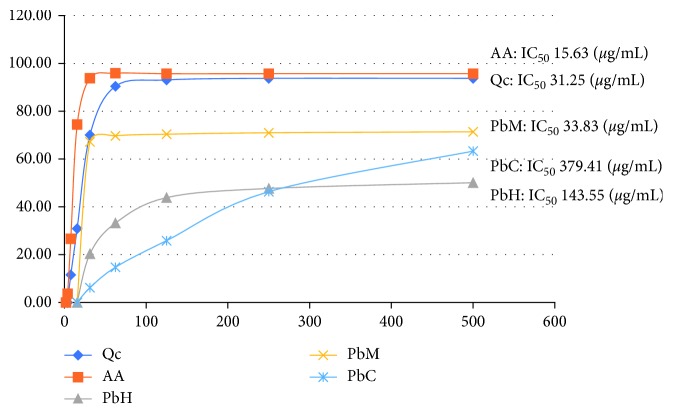
Graph for percent inhibition of DPPH radical of *P. bleo* crude extract and standard at different concentrations.

Scavenging activity of DPPH is based on one-electron reduction which represents the free radical reducing activity of antioxidants. Ascorbic acid (AA) and quercetin (Qc) were used as positive control. All the samples were run in triplicates. The results (Figure 1) showed percentage inhibition of DPPH radical of the *Pereskia bleo* crude extract and standard at different concentrations. The lowest IC_50_ was detected in the methanolic extract (PbM) followed by hexane extract (PbH) and chloroform extract (PbC) with IC_50_ values of 33.83 *µ*g/mL, 143.55 *µ*g/mL, and 379.41 *µ*g/mL, respectively. As the lower IC_50_ value possesses a higher antioxidant activity, the methanol extract has a higher ability to scavenge free radical compared to hexane and chloroform extracts. High antioxidant activity showed by the methanolic extract has a positive relationship with TPC activity, where high TPC gives a high antioxidant capacity due to the linear correlation between the two parameters. Previous studies have shown that the capacity of the antioxidant is highly associated with the total flavonoid content and total phenolic compounds of the plant leaves' crude extract [8–10]. Finding from this study was supported by the findings reported by Sharif et al. [11], where the leaves of the *P. bleo* methanol extract showed the lowest IC_50_ with a value of 68.75 *µ*g/mL. The study also reported the presence of high amounts of phytol, fatty acid, and sterols in the methanolic extract.

### 3.3. Antimicrobial Activity

Antimicrobial activities of *P. bleo* extracts against all four tested bacteria ,namely, *Staphylococcus aureus* (SA), *Streptococcus pyogenes* (SP), *Pseudomonas aeruginosa* (PA), and *Escherichia coli* (EC) were qualitatively and quantitatively appraised by the presence or absence of bacteria based on minimum inhibitory concentration (MIC) and minimum bactericidal concentration (MBC) assays. Based on the MIC values, the methanolic extract displayed strong activities towards Gram-positive bacteria, SA and SP with the MIC value 225 *µ*g/mL, as well as that of against Gram-negative bacteria, PA and EC with the MIC value 450 *µ*g/mL. In addition, the chloroform extract also exhibited strong activities against SA, SP, and EC with the MIC value 225 *µ*g/mL, as well as that of against PA with the MIC value 450 *µ*g/mL. However, the hexane extract demonstrated weak activities towards all four bacteria with the MIC value 1800 *µ*g/mL (Table 2).

The evaluation of antibacterial activity was extended to MBC. From the result, the MBC values were confirmed by the absence of bacterial growth on the nutrients agar streaked from the lowest clear MIC values. Both methanolic and chloroform extracts of *P. bleo* showed strong inhibition activity towards the tested bacteria SA, SP, PA, and EC. Most of the bacterial strains used in this study are related to the food spoilage. For example, SA considered as one of the most common sources of food-borne disease while EC and PA produce toxins that induce human gastroenteritis diseases. Moreover, antibacterial activities studied by Abbdewahab [12] against two bacteria which are *Salmonella choleraesuis* and *Pseudomonas aeruginosa* has reported that methanol and hexane extracts of *P. bleo* show positive result in inhibiting both bacteria. The dichloromethane extract of *P. bleo* also demonstrated a good antibacterial effect against *Staphylococcus aureus* [2, 12]. Besides antibacterial, methanol leaves' extract of *P. bleo* also show great antifungal activity against a plant pathogenic fungus, *Cladosporium cucumerinum* [13]. As the methanolic and chloroform extracts of *P. bleo* showed strong inhibition activity towards the tested bacteria, the present study suggested that this plant extract could have a great potential to be used as food poisoning control and natural preservatives in preserving food for replacing chemical preservative.

## 4. Conclusions

The methanolic and chloroform extracts of *P. bleo* showed strong inhibition activity towards the tested bacteria SA, SP, PA, and EC. The methanolic extract exhibited highest TPC (40.82 mg GAE/g) and possess highest antioxidant activity (IC_50_ value 33.83 *µ*g/mL) compared to chloroform and hexane extracts. Linear correlation among two parameters of the high TPC value which will give great antioxidant capacity are proved in this study where high antioxidant activity demonstrated by the methanolic extract is backed by the high TPC value. Due to the interesting results exhibited by the methanolic and chloroform extracts of *P. bleo*, the study has extended to the isolation of bioactive compounds to uncover its great potential natural source for antibacterial and antioxidant agents.

## Figures and Tables

**Table 1:** Total phenolic content of *P. bleo* hexane, chloroform, and methanolic extracts.

Sample extract	Total phenolic content (mg GAE/g extract)
Hexane	25.20 ± 0.01
Chloroform	31.91 ± 0.01
Methanolic	40.82 ± 0.01

**Table 2:** Inhibitory concentration of MIC activity for crude extracts of *Pereskia bleo.*

Sample extract	SA	SP	PA	EC
Hexane	1800	1800	1800	1800
Chloroform	225	225	450	225
Methanolic	225	225	450	450

*Note*. SA, *Staphylococcus aureus*; SP, *Streptococcus pyogenes*; PA, *Pseudomonas aeruginosa*; EC, *Escherichia coli* (unit in *μ*g/mL).

## Data Availability

No data were used to support this study.
